# Effectiveness of the oral Clonidine as a pre-anesthetic medicine for thyroidectomy surgery; A randomized clinical trial

**DOI:** 10.34172/jcvtr.2023.31680

**Published:** 2023-09-23

**Authors:** Sepideh Sadat Zahedi, Bahman Naghipour, Surur Zahedi, Sahar Zahedi, Seyed Ziaeddin Rasihashemi

**Affiliations:** ^1^Department of Anesthesiology, Imam Reza Hospital, Faculty of Medicine, Tabriz University of Medical Sciences, Tabriz, Iran; ^2^Department of Medicine, Tabriz Azad University of Medical Sciences, Tabriz, Iran; ^3^Department of Thoracic Surgery, Imam Reza Hospital, Faculty of Medicine, Tabriz University of Medical Sciences, Tabriz, Iran

**Keywords:** Total thyroidectomy, Clonidine, α_2_ Agonist, Premedication

## Abstract

**Introduction:**

Hemodynamic disturbance is a common problem in patients undergoing thyroid surgery. It may be due to episodic increases in thyroid hormones (thyroid storm) or stimulation of the carotid sinus baroreflex. The aim of the present study was to investigate effectiveness of the pre-operative oral Clonidine on reducing these hemodynamic changes during total thyroidectomy surgery.

**Methods:**

In a prospective, randomized, double-blind study, 80 patients scheduled for elective total thyroidectomy were randomized to receive either 0.2 mg Clonidine (n=40) or a matched placebo (n=40) orally sixty minutes before entering the operating room. Hemodynamic variables, the duration of surgery, estimated amount of blood loss and the dose of administered remifentanil were recorded for further analysis.

**Results:**

Oral Clonidine was found to be significantly better in maintaining stable hemodynamics compared to the control group. Also, In the Clonidine group, the estimated amount of blood loss (110.4±10 ml vs. 182.2±11.4 mL, *P*=0.04), duration of the surgery (78.26±55.2 min vs. 105.16±61.75 min, *P*=0.027) and administered dose of remifentanil (26.67±6.6 μg vs. 216.2±14.8 μg, *P*=0.01) were also significantly lower than the control group.

**Conclusion:**

Pre-operative administration of 0.2 mg oral Clonidine in patients undergoing total thyroidectomy results in improved perioperative hemodynamic stability and reduced response to perioperative stress.

## Introduction

 Thyroidectomy is a common endocrine surgical procedure.^1,2^ It may have many complications during or after the surgery, including hemodynamic instability, bleeding, laryngeal nerve damage, hypoparathyroidism, and postoperative nausea and vomiting (PONV). Acute intra-operative hypertensive crises may be attributed to episodic surges of thyroid hormones (thyroid storm) or a neurogenic baroreflex phenomenon due to surgical manipulation near the carotid sinus.^3,4^ Thyroid storm in an emergency situations characterized by persistent hypertension, tachycardia, hyperthermia, and end-organ damage.^4^ The neurogenic baroreflex affects cardiac contractility, heart rate and systemic vascular resistance by mechanoreceptors those located around aortic arch and carotid arteries. This reflex exerts its effect via efferent pathways from vasomotor center in the brain.^4^

 Care attention to optimize pre-operative condition can decrease the rate of these crises.^3^ The patient should be maintained in a euthyriod state and any pre-operative hemodynamic or anxiety problems should be treated.^5^ Many pharmacological methods have been used pre or intra-operatively to attenuate these crises with controversial results. Clonidine may be an all in one drug.^6,7^ Many investigations have shown that Clonidine effectively reduces hemodynamic responses to laparoscopic cholecystectomy surgery.^6,8,9^ General anesthesia with tracheal intubation and muscle relaxation is the classic anesthetic technique used for thyroidectomy. Laryngoscopy induced sympathetic stimulation can result tachycardia, hypertension and arrhythmias during anesthesia induction. Similar hemodynamic problems can result during surgical manipulation.^10^ Hemorrhage is usually minimal; however it is a potential risk particularly when thyroid extends retrosternally.^11^

 These potential problems pointed to the need of an optimal pre and intra-operative anesthetic management.^3,6,12,13^ Clonidine is a α2 adrenergic agonist, with central sympatholytic effect. It has an acceptable bioavailability when used orally, with a peak effect of 60-90 minutes and a half-life of 9-12 hours.^7,14,15^ It has been widely used in anesthesia for decades. It is an anesthetic adjunct with sedative, anxiolytic, analgesic and anesthetic sparing effect that has been used to attenuate hemodynamic stress to laryngoscopy and tracheal intubation.^12^ Survey in published studies revealed that there is not any investigation due the pre-operative Clonidine in patients undergoing thyroidectomy surgery. Thus we designed and implemented the present study to investigate the effect of oral Clonidine premedication versus placebo on providing a stable hemodynamic in patients undergoing total thyroidectomy surgery during a randomized clinical trial.

## Materials and Methods

 This prospective double-blind randomized clinical trial was conducted on patients undergoing elective total thyroidectomy surgery, at a teaching based general hospital (Imam Reza Hospital, Iran, Tabriz), from August 2020 to September 2021. The study was approved by the local Institutional Committee of Ethics at Tabriz University of Medical Science (IR.TBZMED.REC.1398.936) and was registered in the Iranian Registry of Clinical Trials center (IRCT20140107016117N3). Written informed consent was obtained from all of the participants. The study was conducted on 80 adult patients, who were candidate of elective surgical total thyroidectomy. The patients allocated in Clonidine or placebo groups (n = 40), randomly. The Clonidine group patients received 0.2mg Clonidine tablet, orally 30-60 minutes before patient transfer to Operation Theater (manufactured by Tolid Darou, Tehran, Iran). In the placebo group, a tablet of the same form as a placebo was prescribed. The investigation treatment prepared by a co-worker nurse and patients and anesthesia and surgical teams were kept blinded to the patients group. Any other pre-operative management and preparation was done as routine activity.

 Any patient candidate of elective total thyroidectomy with the following criteria was eligible to enter the study: age of 18-60 years old, American Society of Anesthesiologist (ASA) physical status I or II, pre-operative thyroid function tests (TSH and free T4) within normal ranges. Patients with any coexisting disease (cardiac, pulmonary, renal, hepatic, cerebral, coagulopathy), diabetes mellitus and drug dependence or allergy to Clonidine were excluded from the study.

 Admitting to operation room, classical monitoring including electrocardiography, noninvasive arterial blood pressure and pulse oximetry were applied and an 18-gauge venous line accessed. After premedication with intravenous (IV) midazolam (30µg/kg) and fentanyl (1 µg/kg) and pre-oxygenation with 100% oxygen, anesthesia was induced with IV Lidocaine (1mg/kg), propofol (2mg/kg) and cisatracurium (0.15mg/kg) and trachea intubated with appropriate cuffed spiral tube (ID size of 8mm in men and 7.5mm in women). Anesthesia was maintained using O2, N2O and isoflurane (1%) mixture and intermittent cisatracurium 0.03mg/kg every 30 minutes. Mechanical ventilation continued by adjusting respiratory rate to maintain an end-tidal carbon dioxide (ETCO2) pressure of 30-35 mm Hg. The mean arterial blood pressure (MAP) was maintained at ± 20% of the basic value by adjusting a remifentanil infusion (0.1-1 µg/kg/min).

 This operation was performed by the same anesthesia and surgical team. Hemodynamic variables were measured and recorded at following time laps: prior to anesthesia induction (basic), 1 and 3min after tracheal intubation, 1min after surgical incision and then every 15 min up on transferring to recovery room. The final record was done in the recovery room before discharge. Any uncontrolled hemodynamic problems were managed by routine interventions (e.g. Atropine, fluid therapy, ephedrine, phenylephrine and trinitroglycerin). Intra-operative blood loss was estimated based on the volume of blood in the suction bottle and the number of bloody gauze pads was also recorded. Total dose of used remifentanil, need for other drugs and duration of surgery.

###  Statistical analysis

 The SPSS statistical software ver. 22.0 (Inc., Chicago, IL IBM Corp) was used to analyze the collected data. The categorical and parametric variables were described as frequency (percentage) and mean ± SD, respectively. Categorical variables were compared between two groups using Chi square or Fisher’s exact test. The distribution of the parametric variables was tested by Kolmogorov–Smirnov test and normal distributed data analyzed by student t test for independent groups. P value < 0.05 was considered statistically significant.

## Results

**Figure 1:**
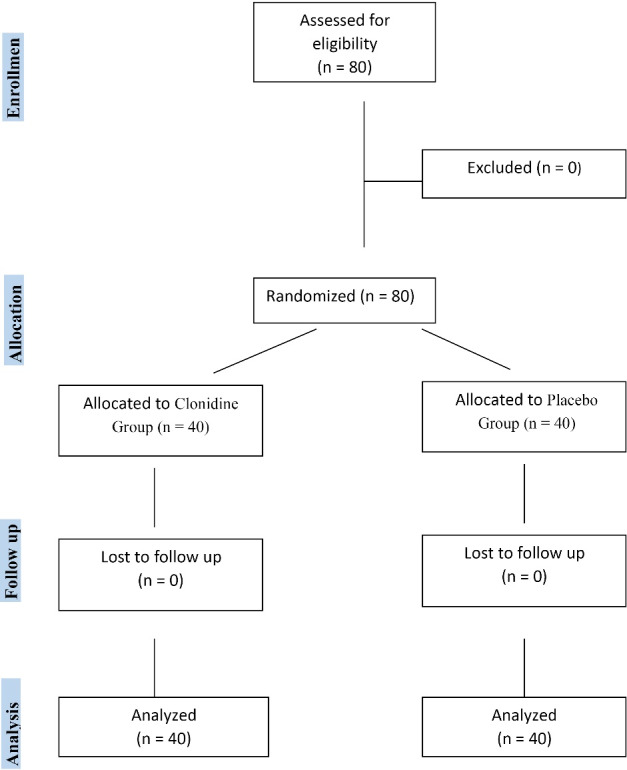
CONSORT follow diagram

 The CONSORT diagram of the study has been illustrated in Figure 1. Any participant was not excluded from the study and there was not any missed to follow up participant.

 Patient’s demographics data are shown in Table 1. There was not any statistically significant difference between the two groups.

**Table 1:** Demographic data of the two groups

**Variable \ Group**	**Placebo group** **(n=40)**	**Clonidine group** **(n=40)**	***P***** value**
Age (year), maen ± SD*	45.3 ± 17.5	42.3 ± 11.6	0.53
Weight (kg), maen ± SD*	64.65 ± 15.32	65.25 ± 12.38	0.89
ASA class (I/II)†	43/37	44/36	0.127
Male/female†	17/63	16/64	0.74

ASA: American society of anesthesiologists physical class, Data were analyzed by student t (*) or chi square (†) tests.

Table 2 shows that comparing to placebo group, the duration of surgery was significantly lower in Clonidine group patients (78.26 ± 55.2 vs. 105.16 ± 61.75 min, respectively, *P* = 0.027). The estimated amount of blood loss was significantly lower in Clonidine group patients (110.4 ± 10 vs. 182.2 ± 11.4 ml, *P* = 0.04). In Clonidine group, the required dose of remifentanil, was significantly lower than placebo group patients (26.67 ± 6.6 vs. 216 ± 14.85 µg, *P* = 0.01).

**Table 2:** presents the duration of the surgery, amount of blood loss and used remifentanil. Duration of surgery, amount of blood loss and used remifentanil were significantly lower in Clonidine group patients

**Variable \ Group**	**Placebo group ** **(n=40)**	**Clonidine group** **(n=40)**	***P***** value**
Blood loss (ml), maen ± SD	182.2 ± 11.4	110.4 ± 10	0.04
Duration of surgery (min), maen ± SD	105.16 ± 61.75	78.26 ± 55.2	0.027
Remifentanil dose (µg), maen ± SD	216.2 ± 14.8	26.67 ± 6.6	0.01

Data were analyzed by student unpaired t-test.

 There was not any need to administer vasodilator, vasopressor and isotropic agents.

 The basal mean arterial blood pressure (MAP) and basal heart rate (HR) were similar in both groups (Tables 3 and 4). Table 3 shows the MAP values at different time laps. At all times after induction of anesthesia until discharge from recovery, MAP values were lower in Clonidine group patients. Table 4 shows the heart rate at different time laps. Similarly to MAP values, at all times HR was lower in Clonidine group patients.

**Table 3:** presents the mean arterial pressure (MAP). There was statistically significant difference between two groups

**MAP**	**Placebo**	**Clonidine**	***P***** value**
basic	101.55 ± 20.8	100.35 ± 14.3	0.77
Post-induction 1th minute	92.9 ± 17.1	83.4 ± 19.2	0.03
Post-intubation 1th minute	92.2 ± 25.5	82.3 ± 18.9	0.013
Post-intubation 5th minute	87.3 ± 17.3	74.5 ± 16.7	0.001
Post incision 1th minute	95.4 ± 19.8	73.2 ± 15.3	< 0.001
Post incision 15th minute	91.7 ± 18.3	72.6 ± 15.5	< 0.001
Post incision 30th minute	83.7 ± 14.6	67.6 ± 14.4	< 0.001
Post incision 45th minute	84.6 ± 13.4	65.1 ± 14.8	< 0.001
Post incision 60th minute	86.5 ± 14.4	65.2 ± 18.0	< 0.001
Post incision 75th minute	86.2 ± 13.4	64.7 ± 12.1	< 0.001
Post incision 90th minute	86.5 ± 14.6	63.4 ± 11.2	< 0.001
recovery	100.2 ± 12.7	76.8 ± 13.3	< 0.001

MAP: Mean arterial pressure, Data were analyzed by student unpaired t-test.

**Table 4:** presents the heart rate during surgery. There was statistically significant difference between two groups

**HR**	**Placebo**	**Clonidine**	***P***** value**
basic	81.2 ± 18.2	83.3 ± 18.4	0.77
Post-induction 1th minute	75.5 ± 14.3	71.7 ± 14.4	0.03
Post-intubation 1th minute	81.8 ± 13.1	70.3 ± 15.5	0.013
Post-intubation 5th minute	77.4 ± 12.9	68.8 ± 12.5	0.001
Post incision 1th minute	78.3 ± 12.5	77.7 ± 14.3	< 0.001
Post incision 15th minute	74.7 ± 11.1	66.7 ± 12.4	< 0.001
Post incision 30th minute	73.2 ± 11.5	65.7 ± 10.7	< 0.001
Post incision 45th minute	71.7 ± 10.1	63.2 ± 9.3	< 0.001
Post incision 60th minute	71.2 ± 10.9	63.3 ± 8.3	< 0.001
Post incision 75th minute	72.4 ± 12.4	62.1 ± 6.8	< 0.001
Post incision 90th minute	76 ± 12.7	60.1 ± 11.5	< 0.001
recovery	87.6 ± 9.8	81.3 ± 24.2	< 0.001
average	77.9 ± 11.8	68.7 ± 13.2	< 0.001

HR: Heart rate, Data were analyzed by student unpaired t-test.

## Discussion

 Total thyroidectomy may be attributed to a lot of common complications during or after surgery including bleeding, laryngeal nerves damage, hypoparathyroidism, hemodynamic instability and postoperative nausea and vomiting (PONV). Acute intra-operative hemodynamic changes may be attributed to episodic surges of thyroid hormones or a neurogenic baroreflex phenomenon due to surgical manipulation near the carotid sinus.^3,4^ Thyroid storm is an emergency situations characterized by persistent hypertension, tachycardia, hyperthermia, and end-organ damage. Anesthetic and surgical teams must prepare and run any activity to perform the surgery in an optimal condition.^3^ Thus the patient should be maintained in an euthyriod state and any hemodynamic or anxiety problem should be treated pre-operatively.^5^ Also many pharmacological methods have been used pre or intra-operatively to attenuate this adverse hemodynamic responses with controversial results, Clonidine may be an all in one drug.^6,7^ Singh et al and Sung et al showed that Clonidine effectively reduces hemodynamic responses to laparoscopic cholecystectomy surgery.^6,8,9^

 In the present study we used oral Clonidine as premedication in purpose of providing a more stable intra-operative hemodynamic during total thyroidectomy. Clonidine using orally, has an excellent rapid absorption with prolonged duration of action that induce a central sympatholytic effect via adrenergic α2 receptors activation. This sympatholytic effect can blunt any hemodynamic stimulation caused by direct laryngoscopy and surgical stimulation. It also induces some analgesic, sedative, anxiolytic, antiemetic and anti-shivering actions in pre, intra and post-operatively.^16,17^ In the present study pre-operative oral Clonidine (0.2 mg) blunted effectively the hemodynamic responses to tracheal intubation and surgical stimulation, reduced intra-operative analgesic requirement and reduced the duration of the procedure probably by providing a more bloodless surgical field.

 Our findings are supported by some studies those investigated efficacy of the pre-operative Clonidine and reported its benefit on blunting hemodynamic responses to laryngoscopy, intubation and surgical stress in thyroid surgeries.^18^

 Reid and Brace described the hemodynamic response to laryngoscopy and intubation due to intense sympathetic discharges caused by stimulation of larynx.^19^ These hemodynamic changes can be detrimental in elderly or hemodynamically compromised patients and induce myocardial ischemia or cerebral hemorrhage.^20-22^ Nishikawa studied the effects of oral Clonidine on the hemodynamic changes associated with laryngoscopy and tracheal intubation and reported that Clonidine attenuated these changes effectively.^23^

 We used Clonidine in dose of 0.2 mg, however other studies has used it in different doses (2-6mcg/kg) but it can be concluded that higher doses does not have any additional advantages comparing to lower doses.^24,25^ Carabine showed that very low doses of intravenous Clonidine (0.625 μg/kg) can attenuate the cardiovascular responses to laryngoscopy.^26^ Clonidine may induce postoperative hypotension.^27^ Also we followed our patients only up to the leaves the recovery until there was not any report of hypotension.

 Clonidine has been used as anesthetic adjuvant and has anxiolytic, analgesic and anesthetic/opioid sparing effects.^7,17^ It affects nociceptive transmission and decreases plasma norepinephrine concentration. Thus it is not surprising that all these effects occurred in our study. Finding of the Wright study supports our study that Clonidine has a potent anxiolytic effect.^28^ In our study remifentanil requirement was significantly lower in Clonidine group patients. Ray et al and Keniya observed similar findings in their study.^24,29^

 Remifentanil is a popular short acting opioid that is used during thyroid surgery. Generally when it is titrated to effect, remifentanil is a safe drug; however in unskilled hands it may induce profound hemodynamic instability.^30^ Clonidine may provide a more safe surgical condition by its opioid sparing effects.^7,31^

 Another finding of current study was a reduced surgical bleeding and duration. The reduced bleeding may be explained by Clonidine pharmacological action. The drug causes vasoconstriction by acting via post-junctional alpha-2 adrenergic receptors and also induces hypotension and bradycardia through its central sympatholytic effect.^32^ The reduction in operative time can be explained by the provision of a clean, blood-free surgical field that facilitates tumor resection.

 In supporting our findings there are several reports that Clonidine can provide a bloodless field during endoscopic sinus surgery, open rhinoplasty or middle ear microsurgery to decrease the mucosal blood flow in animal models.^33-35^ In the past decade the Clonidine congener, dexmedetomidine has been used intra-operatively with a good results of attenuating of the sympathetic response to laryngoscopy and intubation.^29^ Also dexmedetomidine can be a good selection, but Clonidine may be preferred due to its effects in all of pre, intra and post-operative periods. Comparing to dexmedetomidine, Clonidine is a drug of good bioavailability after oral administration, long acting period and rare side effects.^36^ The current study has some limitations. The small sample size, short patients’ follow-up period, studding only on ASA physical class I and II patients, and patients under 60 years old were the limitations.

## Conclusion

 In conclusion pre-operative oral Clonidine 200 mcg is an effective regime of attenuating hemodynamic response to anesthesia and surgical intervention in patients undergoing total thyroidectomy. It also reduces duration of the surgery, estimated blood loss and requirement to remifentanil comparing to placebo.

## Acknowledgments

 We acknowledge the patients for giving their consent in taking part in the study, our nursing in-charge to give drugs in a blinded form and our professional anesthesia care and surgical team.

## Competing Interests

 Authors declare that there is not any conflict of interests about this study.

## Ethical Approval

 The study was approved by the Institutional Committee of Ethics at Tabriz University of Medical Science (IR.TBZMED.REC.1398.936) and was registered in the Iranian Registry of Clinical Trials center (IRCT20140107016117N3). Written and informed consent was obtained from all of the participants.

## Funding

 This study was supported by a fund from the Vice Chancellor for Research of Tabriz University of Medical Sciences.
